# A single early warning signs (SEWS) system for recognizing clinically deterioration outperforms the national early warning score (NEWS) by having a lower activation threshold, broader clinical scope, and faster response time

**DOI:** 10.1016/j.resplu.2025.100947

**Published:** 2025-03-26

**Authors:** Raúl J. Gazmuri, Rebecca Bieber, Calis Lim, Mylene Apigo, Ma Lea Martin

**Affiliations:** a Critical Care Section at the CAPT James A. Lovell Federal Health Care Center, North Chicago, IL, United States; b Resuscitation Institute at Rosalind Franklin University of Medicine and Science, North Chicago, IL, United States; c Chicago Medical School at Rosalind Franklin University of Medicine and Science, North Chicago, IL, United States

**Keywords:** Clinical deterioration, Electronic health record, In-hospital cardiac arrest, National early warning score, Rapid response systems, Vital signs

## Abstract

**Background:**

The National Early Warning Score (NEWS) is a vital-signs point summation system developed to identify patients at risk of adverse events including cardiac arrests, unplanned ICU admissions, and deaths. The points are usually calculated by the Electronic Health Record after charting, recommending local actions and Rapid Response System (RRS) activation when reaching ≥ 7 points. NEWS, however, lacks consistent evidence that it improves outcome and may lead to alarm fatigue. At our institution we operate a Single Early Warning Signs (SEWS) system for RRS activation with a broader range of abnormal signs, without point summation, and bedside assessment within 10 min.

**Methods:**

We analyzed 182 RRS activations using SEWS from July 1, 2022, to August 21, 2023, and compared the activation thresholds and dispositions that would have occurred had NEWS been used.

**Findings:**

At the time of RRS activation using SEWS, only 10 patients (5.5%) had scored ≥ 7 NEWS points. Of the remaining 172 patients, 158 (86.8%) scored 0 to 4 NEWS points considered low risk and 14 (7.7%) scored 5 to 6 NEWS points considered medium risk (*p* < 0.001). Yet, 122 patients (67%) were transferred to a higher level of care including 58 patients (31.8%) to ICU. The median in-hospital cardiac arrest during the reported period was 0.8 per 1000 hospital admissions, which is substantially lower than reported rates.

**Conclusion:**

SEWS operating with a broader clinical scope, lower activation threshold, and faster RRS activation outperformed NEWS markedly reducing in-hospital cardiac arrests.

## Introduction

**Fig. 1:**
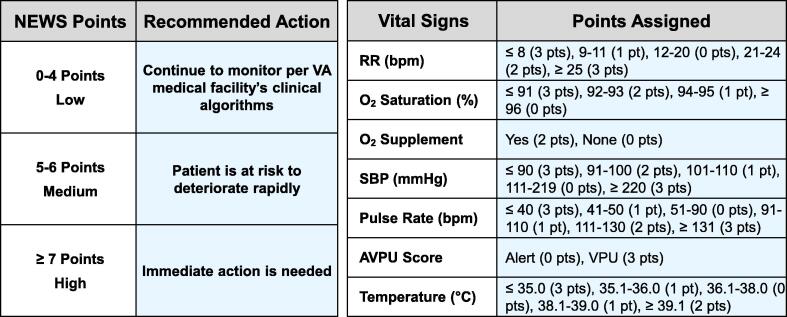
NEWS recommended actions based on total vital-signs (VS) point summation by the Veterans Health Administration: For 0–4 points (Low), NEWS recommends that any single parameter score of 3 in only one category should not drive intervention alone or increase VS frequency. For 5–6 points (Medium), NEWS recommends increasing vital signs to every 2 h for three times (next 6 h total) and reassess NEWS with each vital sign set, contact VA healthcare provider, site specific nursing resources, and follow local VA medical facility’s clinical algorithms. For ≥ 7 points (High), NEWS recommends notifying the Charge Nurse, primary team immediately, and activating the Rapid Response System (RRS) Team. RR = Respiratory Rate; SBP = Systolic Blood Pressure; AVPU = A: Alert, V: Verbal, P: Pain, U: Unresponsive, to assess level of consciousness.

The National Early Warning Score (NEWS) is a vital-signs point summation system widely utilized to identify in-hospital patients at risk of adverse events, including cardiac arrests, unplanned ICU admissions, and deaths.^1^, ^2^, ^3^, ^4^ Points are added based on deviations of respiratory rate, O_2_ saturation, need of O_2_ supplementation, systolic blood pressure, pulse rate, level of consciousness, and body temperature from normal values. According to the total point summation, the risk of further deterioration is rated as low (0–4 points), medium (5–6 points), or high (7 or more points). Specific actions are recommended as shown in Fig. 1, with activation of the institution’s Rapid Response System (RRS) when high-risk of further deterioration is reached (i.e., 7 or more NEWS points). NEWS was selected to be included in the new Federal Electronic Health Record (EHR) throughout the VA enterprise. However, despite the ability to identify patients at risk of adverse events, NEWS and other Early Warning Systems (EWS) have not demonstrated consistent reduction of these adverse events.^2^, ^3^, ^5^ A Cochrane Systematic Review published in 2021^3^ concluded that based on currently available evidence, EWS and RRS may lead to little or no difference in hospital mortality and unplanned ICU admissions. The International Liaison Committee on Resuscitation acknowledged the findings of the Cochrane Systematic Review in a special report published in 2023 titled “Ten Steps Toward Improving In-Hospital Cardiac Arrest Quality of Care and Outcomes,”^6^ yet they also recognized that EWS and RRS are still recommended by several key organizations.^6^

**Fig. 2:**
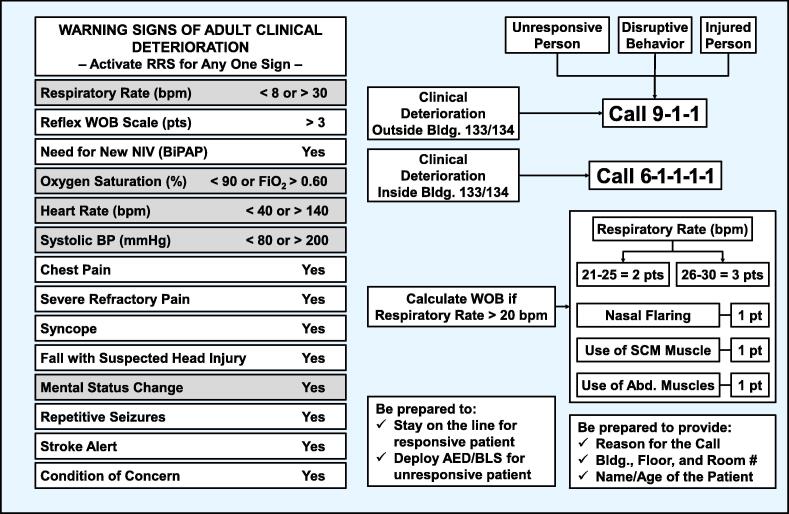
Depiction of our SEWS-based RRS depicting 14 warning signs including our work of breathing (WOB) Scale. Only 5 of the 14 SEWS have comparable signs in NEWS, while temperature is not included in SEWS. NIV = Non-invasive Ventilation; BiPAP = Bilevel Positive Airway Pressure.

Over the span of more than two decades, the ICU team at our institution led the developed of a system for early recognition of clinical deterioration based on Single Early Warning Signs (SEWS) that is currently embedded in our culture of safe patient care. The initial approach consisted of a list of signs given to nurses with instructions to contact a critical care fellow for any single abnormal sign. The system evolved into a Medical Emergency Response System inspired by the work of deVita *et al*.^7^ and subsequently it was integrated into the broad institutional RRS that includes responses to various other emergencies (Fig. 2). For clinical deterioration, our RRS is operated from the ICU without needing a separate team. Once clinical deterioration is recognized, a call is made to the ICU through a dedicated phone answered by an ICU nurse who gathers basic information and activates the response announcing the location through an overhead announcer. The response involves bedside assessment by an ICU nurse and an ICU resident within < 10 min followed by discussion with the ICU attending for disposition. The list of SEWS is regularly revised and updated based on activation frequency of individual signs, new clinical events, and more recently with the inclusion of a work of breathing scale develop at our institution.^8^ Revisions are followed by staff education and updates of posters and pocket cards. We herein report a comparison between the performance of our SEWS system, which includes 14 early warning signs (Fig. 2), with the performance of NEWS (Fig. 1), had it been used instead.

## Methods

We analyzed 182 RRS activations using SEWS from July 1, 2022, to August 21, 2023, and compared the activation thresholds and dispositions that would have occurred if NEWS had been used. Because NEWS assigns points contingent on the magnitude of the deviation from normal within a specific warning sign (as shown in Fig. 1), for our analysis, we assigned the maximal NEWS point to the abnormal vital sign that corresponded to the sign or signs that activated our RRS. For example, if the activation resulted from an O_2_ Saturation < 90% or FiO_2_ > 0.6 we would assign 5 NEWS points encompassing two criteria (i.e., O_2_ saturation and O_2_ supplement), therefore biasing in favor of NEWS.

Statistical differences in NEWS points among the three categories (i.e., low, medium, and high) were analyzed using the Kruskal-Wallis ANOVA on ranks. We also reported the incidence of in-hospital cardiac arrests (IHCA) during the observation period.

## Results

**Fig. 3:**
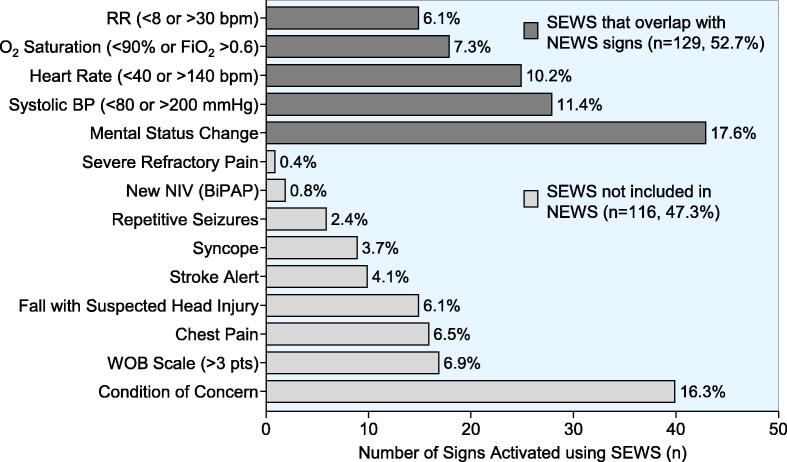
Rapid Response System (RRS) activations (n = 182) triggered by 245 SEWS including 45 activations (25.7%) for more than one concurrent SEWS. Only 129 (52.7%) SEWS activations had equivalent NEWS signs and 116 (47.3%) SEWS activations had no equivalent sign in NEWS, with Condition of Concern (not available in NEWS) being the second most frequently activated SEWS after Mental Status Change. RR = Respiratory Rate; BP = Blood Pressure; NIV = Non-invasive Ventilation; BiPAP = Bilevel Positive Airway Pressure; WOB = Work of Breathing.

The 182 RRS activations were responsive to 245 SEWS with 45 (24.7%) activations including more than one SEWS. As shown in Fig. 3, only 52.7% of the activations had an equivalent sign in NEWS with 47.3% of the activations triggered by signs not available in NEWS, including “Condition of Concern” which was the most frequent activation sign after “Mental Status Change.” The disposition after the 182 RRS activations resulted in 122 patients (67%) transferred to a higher level of care with 58 (31.9%) admitted to ICU, mostly from the general medical ward. Another 57 patients (31.3%) were transferred to the Emergency Department for evaluation, mostly from our nursing home. Only 59 patients (32.4%) remained at their site of origin.

**Fig. 4:**
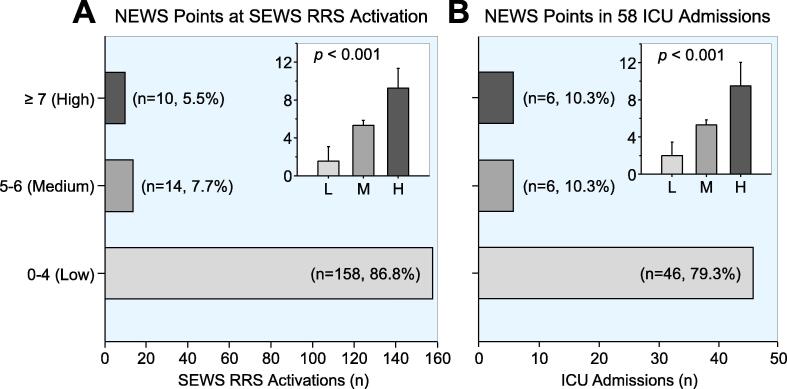
**A**) Distribution of NEWS point-based categories at the time of 182 RRS activations with 86.8% scoring a Low NEWS category. **B**) Distribution of NEWS point-based categories of 58 patients admitted to ICU after RRS activation with only 10.3% scoring a High NEWS category. The inserts depict the points ± SD withing each NEWS category with the overall difference calculated using ANOVA on ranks.

Fig. 4A shows that using NEWS, only 10 of the 182 patients (5.5%) would have reached the ≥ 7-point threshold for RRS activation. Most of the patients (158 patients, 86.8%) scored between 0 and 4 NEWS points at the time of RRS activation, which is defined as low risk for further deterioration. Fig. 4B shows 58 patients admitted to ICU after RRS activation. Only 6 (10.3%) of these patients reached the NEWS threshold for RRS activation and 46 of them (79.3%) were classified as low risk despite the decision for ICU admission, which would have delayed evaluation by the RRS team until further clinical deterioration.

During the period of our analysis, there were three IHCA for 3554 hospital admissions to areas covered by our RRS resulting in an incidence of 0.8 per 1000 hospitalizations. The cardiac arrests occurred in the ICU consequent to disease severity and not to failed recognition of clinical deterioration.

## Discussion

The key distinctive features of SEWS compared to NEWS are (1) activation after recognition of a single abnormal sign without need for point-summation, (2) inclusion of several other signs without equivalent NEWS signs, and (3) immediate RRS activation upon recognition of the abnormal sign. The combination of these three key features resulted in a substantially lower threshold for RRS activation with broader clinical scope and in real-time enabling early diagnosis and treatment of the condition responsible for the clinical deterioration.

NEWS and other point-summation systems typically rely on the EHR for automatic score calculation. Inherent delays associated with EHR charting, multiple competing healthcare tasks, and the high threshold with narrow scope for RRS activation likely explains the failure to demonstrate reduction on the adverse outcomes able to anticipate.^3^, ^9^ The high NEWS threshold for RRS activation with local actions for lower scores are intended to minimize alarm fatigue. Yet, alarm fatigue still occurs, and healthcare providers become desensitized to frequent alerts failing to act when appropriately needed.^10^, ^11^, ^12^

In addition, adequate implementation of NEWS and other EWS depends on adequate training and protocol adherence.^13^ When staff are overwhelmed and/or incompletely trained, the system may fail to activate the RRS in a timely manner.^14^ Systemic issues of staff shortages and time constraints only exacerbate the challenges, hindering NEWS’s ability to be reliable and consistently identify patients experiencing clinical deterioration.^15^, ^16^

A system of activation based on SEWS has the advantage of simplicity, prompting RRS activation in real time without reliance on the EHR and the complexity of point-summation to achieve the activation threshold. Although, such a system might at first suggest low specificity with unnecessary activations leading to reluctance of RRS activation, in our study, two thirds of the patients were transferred to a higher level of care which enabled early diagnosis and treatment curtailing the progression of clinical deterioration. In addition, our SEWS system was associated with an IHCA of 0.8 per 1000 hospitalizations, substantially lower than the IHCA reported by Bradley *et al*.^5^ in 101 VA hospitals ranging from 1.4 to 11.8 per 1000 hospitalizations. A similar IHCA incidence ranging from 1.2 to 10.0 per 1000 hospitalizations has been reported in non-VA hospitals in the USA and abroad in industrialized countries.^17^, ^18^, ^19^, ^20^

An important aspect of our SEWS system is the integration within a culture of patient safety that is embraced and supported by healthcare providers. Our system was organically developed and evolved over more than 20 years. In addition, our system has undergone multiple adjustments in response to clinical events and requests by healthcare personnel, providing a sense of ownership. Another key feature is its development and operation by the ICU team. Because failure to recognize clinical deterioration will end with adverse events requiring ICU interventions, such as unplanned ICU admissions, emergency intubations, and cardiac arrests, the ICU team recognizes the value of their involvement in a system for early recognition of clinical deterioration in all hospitalized patients. In addition, our system includes education and training of all personnel involved and performance monitoring for quality improvement.

Adoption of a similar system in environments with a larger patient load and less critical care resources might be challenging. Yet, “bending the curve” of further clinical deterioration by early recognition and improving outcome using fewer healthcare resources might be a cost-effective strategy worth investigating in such settings.

## Conclusions

Timely recognition of clinical deterioration with prompt effective intervention remains a challenge. Our study suggests that systems that are simple, operate in real-time, have low threshold for activation, and maintain a broad scope operating within a culture of patient safety and sense of ownership are likely to be more effective than current point-summation systems inserted in the EHR.

## CRediT authorship contribution statement

**Raúl J. Gazmuri:** Writing – review & editing, Writing – original draft, Supervision, Formal analysis, Project administration, Methodology, Conceptualization. **Rebecca Bieber:** Writing – review & editing, Investigation. **Calis Lim:** Writing – review & editing. **Mylene Apigo:** Supervision, Investigation, Formal analysis, Data curation, Conceptualization. **Ma Lea Martin:** Investigation, Data curation.

## Declaration of competing interest

The authors declare that they have no known competing financial interests or personal relationships that could have appeared to influence the work reported in this paper.
